# Translating the “Banana Genome” to Delineate Stress Resistance, Dwarfing, Parthenocarpy and Mechanisms of Fruit Ripening

**DOI:** 10.3389/fpls.2016.01543

**Published:** 2016-10-26

**Authors:** Prasanta K. Dash, Rhitu Rai

**Affiliations:** ICAR-National Research Centre on Plant BiotechnologyNew Delhi, India

**Keywords:** Banana/plantain, evolution, structural genome, disease resistance, parthenocarpy, dwarfism, biotic and abiotic stress, comparative genomics

## Abstract

Evolutionary frozen, genetically sterile and globally iconic fruit “Banana” remained untouched by the green revolution and, as of today, researchers face intrinsic impediments for its varietal improvement. Recently, this wonder crop entered the genomics era with decoding of structural genome of double haploid Pahang (AA genome constitution) genotype of *Musa acuminata*. Its complex genome decoded by hybrid sequencing strategies revealed panoply of genes and transcription factors involved in the process of sucrose conversion that imparts sweetness to its fruit. Historically, banana has faced the wrath of pandemic bacterial, fungal, and viral diseases and multitude of abiotic stresses that has ruined the livelihood of small/marginal farmers’ and destroyed commercial plantations. Decoding structural genome of this climacteric fruit has given impetus to a deeper understanding of the repertoire of genes involved in disease resistance, understanding the mechanism of dwarfing to develop an ideal plant type, unraveling the process of parthenocarpy, and fruit ripening for better fruit quality. Further, injunction of comparative genomics will usher in integration of information from its decoded genome and other monocots into field applications in banana related but not limited to yield enhancement, food security, livelihood assurance, and energy sustainability. In this mini review, we discuss pre- and post-genomic discoveries and highlight accomplishments in structural genomics, genetic engineering and forward genetic accomplishments with an aim to target genes and transcription factors for translational research in banana.

## Introduction

**FIGURE 1:**
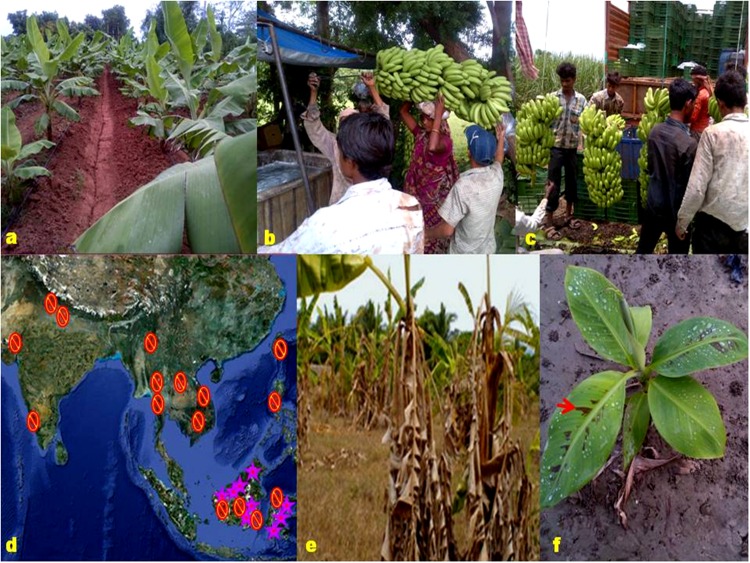
**Banana cultivation is one of the major land uses in Asia and Africa not only for nutritional requirement but also a key household source of income for marginal farmers.** The banana genome sequence, comprising a high-resolution map of genes, repetitive elements and variation (single-nucleotide polymorphisms, INDELs) will be leveraged by researchers/breeders to construct high-density, well-placed marker sets. Further, marker-assisted selection will allow breeders to sort through thousands of recombinants to select plant type that contain only the beneficial gene(s) and remove linkage drag. **(a)** A commercial banana plantation in northwest India. **(b)** Single bunch of banana is handpicked and carefully washed to minimize the bruises and marketable loss to the fingers. **(c)** Harvest-mature looms are transported for ripening to nearby banana industry depicting intricate association of livelihood for farmers, traders, distributors, and industry. **(d)** Origin and distribution of bananas representing primary center of origin (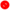) that geographically overlaps from northeast India, Thailand, Myanmar, Bangladesh to other Polynesian regions and secondary center of diversity 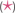 covering peninsular Malaysia and Indonesia. **(e)** A banana plantation devastated by Panama wilt caused by *Fusarium oxysporum* sp. *cubense race 4*. **(f)** Typical leaf-spot symptoms of black sigatoka disease in young leaves of banana caused by *Mycosphaerella fijiensis*. Source: **(d–f)** from Google (www.google.com).

Banana is a unique and iconic fruit among global consumers. The fruit has numerous health benefits such as subsiding appetite, sugar craving, fighting obesity, improving muscle endurance, and reducing bad cholesterol in humans. Along with its related clade plantain, it is grown as a cash crop^1^ around the world with more than 85% for local consumption (Sharrock and Frison, 1998). Predominantly, it is cultivated in tropical and sub-tropical countries (van Asten et al., 2011) and is an important source of livelihood of marginal farmers of Asia and South-east Asia (**Figure 1a,b,c**) (Sreedharan et al., 2013). It ranks second to mango as the most consumed fruit in the world and is a source of food security in many developing countries (Martin et al., 2013). This fruit has a unique yellow peel and sweet flesh which makes it popular as a part of healthy diet in industrialized countries as well. The canoe shaped fruit is usually fried, roasted, or boiled when green, while special fruit types of the East African highland plantain cultivar are preferentially brewed. It ranks next to rice, wheat and maize (Perrier et al., 2011) in terms of its importance as source of staple starch with global annual production of 106 MT in which Asia contributes 60 MT (WBF: Statistics and data, 2012).

## Banana Biology, Origin and Evolution

Taxonomically, bananas and plantains are monocotyledonous plants in the genus *Musa* (Family: *Musaceae*; Order: *Zingiberales*) that comprises “Eumusa” with 22 chromosomes and “Australimusa” with 20 chromosome sets (Perrier et al., 2009, 2011). Though, botanically it is a monocotyledonous perennial herb (Uma et al., 2008), the presence of the unique tall order pseudo-stem apparently attributes it the tree phenotype (approximately 2.0–3.5 m). However, absence of typical traits of tree stems such as lignification and secondary thickening of the pseudo-stem differentiates them from trees (Wu et al., 2016).

Banana is considered to have originated in Asiatic region (Simmonds, 1962) with South-east Asia covering India to Polynesia as the primary center of origin; while the tropical rainforest of Malaysia–Indonesia is considered as the center of diversity with occurrence of diverse endemic wild banana species (**Figure 1d**). As per the International Classification System (ICS) *Musa* has five major sections, viz., *Eumusa, Rhodochlamys, Callimusa*, *Australimusa* and *Incertae sedis.* Of these sections, *Eumusa* contributes to the wide diversity for wild and cultivated types. There are 11 wild species reported across the globe of which six species are prevalent in India. *M. acuminata* and *M. balbisiana* are the major progenitors of present day bananas and have a wider distribution throughout the banana growing states including Andaman and Nicobar Islands.

Plantains and bananas (*Musa spp.* section Eumusa) originated from intra and inter-specific hybridization between two wild species namely *M. acuminata* Colla (A genome) and *M. balbisiana* Colla (B genome). Polyploidy and hybridization have given rise to a number of diploid, triploid, and tetraploid clones with different combinations of A and B genomes like AA, AB, AAA, AAB, ABB, AABB, AAAB, ABBB (Saraswathi, 2011; Vanhove et al., 2012; Davey, 2013). Dessert and highland bananas are classified mainly as AAA, plantains are AAB, and cooking bananas are ABB. The first and most crucial step in evolution of edible bananas was the development and selection for parthenocarpy and seed sterility in *M. acuminata*, giving rise to the edible diploid (AA) cultivars. It is believed that chromosome restitution at meiosis in *M. acuminata* gave rise to edible triploid AAA (*acuminata*) bananas. Though not clear, using variation in anthocyanin pigments as a marker, it is assumed that three different subspecies such as *malaccensis*, *banksii*, and *zebrina* (Horry and Jay, 1990) were involved in the evolution of triploid edible bananas.

## Banana Diversity and Use

Till 1970, the delicious ‘Gros’ Michel or ‘Big Mike’ bananas were the globally accepted varieties for consumption. However, after out-break of the “Panama disease,” they were replaced by the yellow Cavendish variety (Heslop-Harrison and Schwarzacher, 2007) which were resistant to Panama wilt. Currently, more than 90% of the bananas sold in the market are of Cavendish type. ‘Robusta’ (AAA), a Cavendish cultivar of banana, ripens without changing to yellow (green ripe) and has good shelf-life with mild taste and mushy texture of typical Cavendish. ‘Nendran’ type banana (AAB) is preferred for cooking and making chips while ‘Peyan’ (ABB) is a dessert banana. ‘Poovan’ (AAB) and ‘Njalipoovan’ are sweet dessert banana with small fingers, thin skin and delicate flavor but exhibit poor keeping quality thus reducing the marketable value. Currently, a handful of other prized types are available such as ‘red’ (AAA) - a delicious banana that has a sweet taste and creamy texture. The ‘Burro’ varieties grown in Mexico have a sweet-and-sour taste but are stubbier and fatter than the Cavendish type. ‘Manzano’ variety is native to Central and South America and possesses a firmer texture than Cavendish with complex scent and taste, marked by a strong tart-apple aroma that gives sweetness after chewing. Another variety named as ‘Baby’ for their miniature size (Malaysian origin) and is sweet in taste after complete ripening. A different clade called ‘Plantain’ is usually cooked for consumption and serves as an economical but delicious substitute for potatoes/rice in Latin American cuisines.

## Banana Genomics

Genetically, banana is a triploid crop and comprises of both A (*M. acuminata*) and B (*M. balbisiana*) genomes. The triploid genetic constitution of banana posed a huge challenge to the sequencing and assembly of its genome. Therefore, a double haploid (PH-Pahang) *M. acuminata* was generated and sequencing was accomplished in 2012 by a team of researchers (D’Hont et al., 2012) under the framework of Global Musa Genomics Consortium. The genome size of *Musa* ranges from 554 megabase (Mb) in *M. balbisiana* to 523 Mb in *M. acuminata*. The genome is two/three times larger than the genomes of cultivated rice (*Oryza sativa*, 250 Mb) or *Arabidopsis* (150 Mb) but still is much smaller than the *Triticeae* cereals such as wheat (17,000 Mb), barley (5,500 Mb) and rye (9000 Mb; D’Hont et al., 2012). Nonetheless, the 523 Mb genome of banana was precisely sequenced with a combination of Sanger, 454 and Illumina technologies and 90% assembled into 7000 scaffolds with contiguity *on par* with other plant genomes (1.3 Mb scaffold N50). Further, 70% of the assembly anchored to 11 Pahang chromosomes with 250 scaffolds covering 92% of the annotated genes. Sequencing information further revealed that banana genome contained approximately 36,000 genes which were mostly located in the distal-end of the chromosomes. It was also observed that like most plant genomes (Singh et al., 2012; Varshney et al., 2012; Wang et al., 2012), the *Musa* genome consists of repetitive DNA (about 50%) comprising of transposable elements.

Interestingly, the number of transcription factors (TFs) identified in *Musa* (3,155) is highest among all sequenced plants. Of these, 759 TFs were specific to banana and belonged to the MYB and AP2/ERF transcription factor families that plays a role in fruit architecture and ripening (D’Hont et al., 2012). Expression profiling study in response to ethylene treatment identified a cassette of transcription factors (597) speculated to contribute to the unique climacteric character of ripening in banana. Genes encoding cell-wall modifying enzymes and β-amylase were found to be up-regulated in contrast to three starch synthase genes which were down regulated during ripening. In addition, two vacuolar invertase genes involved in sucrose conversion, work in reverse tandem to maintain soluble sugar homeostasis that imparts sweetness to ripening bananas.

## Disease Epidemics and Resistance in Cultivated Banana

Cultivated bananas are propagated through vegetative suckers and this clonal method of propagation makes banana particularly vulnerable to diseases. Historically, Panama disease (**Figure 1e**) caused by the fungus *Fusarium oxysporum* sp. *cubense* has inflicted major devastation to the banana plantations (Moore et al., 1995). A virulent form of this pathogen, ‘Tropical Race 4,’ has caused substantial losses in Asia and other regions of the world to both subsistence farmers and commercial growers (Hwang and Ko, 2004). Exploring for genetic resistance in banana germplasm has led to the identification of a number of varieties (Ortiz, 1995) that could be potentially utilized as resistance gene donors for breeding or gene transfer programs. However, the genetically sterile nature of banana is an impediment to resistance breeding. Therefore, utilizing forward genetics tool of induced mutagenesis, a resistant cultivar of Rasthali (Silk, AAB) has been developed to combat fusarium wilt (Saraswathi et al., 2016). The putative resistant plants showed promising results with completely clean corm (disease score 1) that had no vascular discoloration (zero disease manifestation). Further, the double haploid Pahang, used for genome sequencing, was found to be highly resistant to *F. oxysporum* race-4. Functional genomics studies on Pahang would unravel the genes underlying this inherent resistance.

Another fungal disease of banana which has acquired importance is black Sigatoka leaf spot disease (**Figure 1f**), caused by *Mycosphaerella fijiensis* which results in more than 50% crop losses (Ferreira and Silva, 2004). A triple gene construct from heterologous sources viz.(i) endochitinase gene *ThEn*-*42* from *Trichoderma harzianum*, (ii) grape stilbene synthase (*StSy*), and (iii) superoxide dismutase *Cu, Zn*-*SOD* from tomato were used to transform Grand Nain banana (Vishnevetsky et al., 2011). Repeated field trials of this transgenic exhibited tolerance not only to Sigatoka but also to ‘Gray mold/ blossom blight’ – another fungal disease of banana caused by *Botrytis cinerea*.

Viruses form a minority amongst the pathogens aﬄicting banana (Hull, 2002). However, banana streak virus (BSV) formed an extreme case of parasitism before its genome sequence uncovered the molecular facts. Genome sequence assembly data allocated the endogenous viral sequences integrated at 24 loci spanning 10 chromosomes in Pahang genome. Molecular characterization identified them to belong to badnavirus group that differed from the endogenous BSV species (eBSV) found in *M. Balbisiana*, which are known to be capable of vertical transmission. Further, it was observed that these viral sequences in banana genome have lost their original form and integrity and, therefore, can not seemingly form free infectious particles leading to disease. Conclusion from recent genomic study refuted the earlier understanding that integrated viral elements in banana genome are capable of expression and can give rise to *de novo* viral infection (Harper et al., 2002; Iskra-Caruana et al., 2010) leading to disease pandemism.

## Mechanism of Dwarfing in Banana

Commercially cultivated bananas are tall order plants measuring more than two meter in height. The taller canopy makes them vulnerable to high velocity wind, low/high cyclonic typhoons that break the pseudo-stem and ushers irreparable loss to commercial plantations. Therefore, dwarf variety of banana is the desirable ideotype to reap the advantages of efficient agronomic procedures like high density planting and timely cultural practices. Genetically, it has been shown that dwarfism in plants is associated with the reduction of bio-available gibberellic acid (GA). The famous high-yielding semi-dwarf variety of rice (IR8) which ushered in the ‘Green Revolution’ was accomplished due to a mutation in the *sd1* gene. This gene is involved in the biosynthesis of gibberellin and the mutation attenuates the GA biosynthesis there by making the rice plant semi-dwarf. Thus, identification of dwarfism related genes in banana was of paramount importance. Recently, a mutant of ‘Williams’ variety of banana was developed (Chen et al., 2016) that exhibited reduced height (approximately 1.7 m) and their pseudo-stem showed better mechanical strength compared to its progenitor. Using the information from banana genome, it was elucidated that gibberellic acid plays a pivotal role in its dwarfism. Genome-wide screening using contrasting banana genotypes further revealed that 36 genes are involved in GA metabolism in banana. While dwarf banana had differential expression of genes involved in GA homeostasis, the young fruits were observed to be the sites of GA metabolism that contribute to fruit length. Identification of GA metabolism genes will expedite application of molecular techniques such as CRISPR/CAS9 (Cong et al., 2013; Li et al., 2013) to tinker GA homeostasis to develop dwarf banana.

## Parthenocarpy

Banana garners huge demand in human consumption due to its seedless fleshy fruit. The ability of plants to bear seed-less fruit (without pollination and fertilization) is due to the phenomenon known as “Parthenocarpy.” Additionally, female sterility is also hypothesized to be the cause of parthenocarpy in many fruits including banana (Dodds and Simmonds, 1948; Simmonds, 1953, 1962). Parthenocarpy in banana is believed to be the manifestation of A genome (Simmonds, 1962). Growth promoting phytohormones such as auxins, gibberellins, cytokinins and absiscic acid are also reported to be involved in parthenocarpy in melon (Talon et al., 1992) and tomato (Fos et al., 2000; Ding et al., 2013). Though intrinsic mechanism of natural parthenocarpy in plants is still unresolved, it is observed that natural parthenocarpic fruits such as banana (Khalifah, 1966) and mandarin (Talon et al., 1990, 1992) record increase in auxin and gibberellins level while levels of ABA declines (Talon et al., 1990). Genetically, parthenocarpy may happen due to a single partially dominant gene as in cucurbits (Kim et al., 1992; Menezes et al., 2005) or due to a single recessive gene as in sweet pepper (Tiwari et al., 2011) or due pleiotropy of major and minor genes as in brinjal/eggplant (Miyatake et al., 2012). In case of banana, parthenocarpy is hypothesized (Sardos et al., 2016) to be due to a major dominant gene (*P* or *P1*) interacting with minor ones (Simmonds, 1953). With decoding of structural genome of banana, discovery of the precise mechanism of parthenocarpy will give a boost to quality fruit production in commercial plantation.

## Banana Ripening

Ripening in fruits is a natural phenomenon by which they taste sweeter and gain palatability. Biochemically, it begins with concomitant increase in acidity till a level (brix-acid ratio) when fruits taste/smell tarter. Being a climacteric fruit, ripening in banana is an important phenological event. Edible cultivated bananas are triploids with AAA, AAB and ABB genomic composition of which the A-genome (*M. balbisiana*) has been associated with banana pre-harvest development and post-harvest ripening (Davey, 2013). Cultivars possessing more of A-genome exhibit high yield, long fingers, and long-term storage (Hu et al., 2015) an attribute of best fruit quality. Post-genomic research has strongly indicated role of the bZIP transcription factors in fruit development process in many crops (Levi et al., 2006; Costa and Alba, 2010; Rohrmann et al., 2011). Recently, extensive involvement of bZIPs and WRKY in fruit development and post-harvest ripening has been demonstrated (Goel et al., 2016; Hu et al., 2016a) by comparing its expression pattern at different stages of banana development. The high expression levels of MabZIPs in fruit developmental and ripening is postulated to control the fruit quality of banana. Another transcription factor MaDEAR1 (repressor) is reported to be negatively involved in cell wall modification and softening of banana (Fan et al., 2016). With development of suitable transformation/regeneration techniques (Uma et al., 2011, 2012) these TFs will be targets of modulation in banana for best quality looms.

## Abiotic Stress Tolerance

Due to inherent rapid rate of growth, shallow root system and permanent green canopy, banana requires abundant supply of water (van Asten et al., 2011) for optimum yield potential. The B-genome of banana has been the target for breeding programs (Davey, 2013; Hu et al., 2015) as it imparts intrinsic tolerances to both biotic and abiotic stresses. In *M. balbisiana* the B-genome imparts (i) resistance to infection of *Xanthomonas* and (ii) tolerance to drought/water stress (Tripathi et al., 2007; Vanhove et al., 2012; Davey, 2013). At molecular level, it has been reported that multiple bZIP transcription factors are involved in imparting tolerance against cold, drought and salt stress in banana (van Asten et al., 2011), beans (Rodriguez-Uribe and O’Connell, 2006), brachypodium (Liu and Chu, 2015) and rice (Liu et al., 2012). In fact, the cultivars containing the B-genome has been reported to exhibit higher tolerance to abiotic stresses (Hu et al., 2015). Gene expression analysis in banana showed 74% bZIP genes had increased transcription levels under cold, drought, and salt stresses. Commensurate increase in transcription levels was also observed in other plants such as cassava (Hu et al., 2016b), *Brachypodium distachyon* (Liu and Chu, 2015) and maize (Wei et al., 2012). Post-genomic discovery of bZIPs (Hu et al., 2016a), microRNAs (Chai et al., 2015) along with key metabolites (Pandey et al., 2016) is expected to expedite the molecular breeding process by ushering in, understanding the role, these transcription factors in signal transduction, developmental processes and responses to abiotic stress in banana.

## Conclusion and Future Perspective

Banana has remained practically untouched by the ‘Green Revolution’ because of the difficulties encountered in conventional breeding programs in this genetically sterile triploid crop. *Inter alia*, being the most consumed fruit banana has immense capability to contribute to food security through higher and stable yields. It has been a challenge for banana breeders to produce disease resistant polyploid hybrids through genetic recombination. Like other crops (Dash et al., 2014), availability of structural genome information has opened up vistas to saturate the genome with molecular markers which would enable banana researchers to select good plant type at an early stage in commercial plantation. Structural genomics of banana has further delineated molecular mechanisms of dwarfing, imparting disease resistance, role of phyto-hormones in parthenocarpy and pivotal role of transcription factors in formation of this iconic fruit. Now, it will provide impetus to *Musa* researchers to apply functional genomics and comparative genome analysis to identify, clone, and characterize genes responsible for key agronomic traits as well as biotic and abiotic stress tolerance. As of today, the mechanism of action and the inter-connection of various signaling cascades remain largely unknown in banana. Functional analysis to unravel regulatory details small-, micro-, and si-RNAs and their role in the stress responses and hormone signaling will provide useful information for translational research in banana. As our molecular understanding advances, biotechnological interventions such as gene discovery, genetic engineering, targeted transformation/regeneration, and progressive bioinformatic tools (Martin et al., 2016) coupled with validated markers (Backiyarani et al., 2013) will accelerate breeding and super-domestication of banana will be a reality soon.

## Author Contributions

PD planned, collected information, organized, and prepared the manuscript. RR designed and contributed for writing and reviewing the manuscript.

## Conflict of Interest Statement

The authors declare that the research was conducted in the absence of any commercial or financial relationships that could be construed as a potential conflict of interest.

## Footnotes

1 http://www.cgiar.org
